# Chromium-Decorated Double-Walled Carbon Nanotubes for Preferential Room-Temperature Xylene Sensing

**DOI:** 10.3390/ma19204378

**Published:** 2026-10-18

**Authors:** Tlotlo Cassandra Setlhare, Atlang Gild Mpolokang, Emmanuel Flahaut, Martin Figueiredo, George Chimowa

**Affiliations:** 1 Department of Physics and Astronomy, Botswana International University of Science and Technology, Boseja Ward, Palapye Private Bag 16, Botswana; st19100123@biust.ac.bw (T.C.S.); ma19100109@biust.ac.bw (A.G.M.); 2 Université de Toulouse, Toulouse INP, CNRS, CIRIMAT, Toulouse, France; emmanuel.flahaut@utoulouse.fr (E.F.); martin.figueiredo@utoulouse.fr (M.F.)

**Keywords:** double-walled carbon nanotubes, chromium(II) chloride, endohedral filling, chemiresistive gas sensor, xylene sensing, BTX discrimination, volatile organic compounds

## Abstract

Room-temperature discrimination of closely related aromatic volatile organic compounds remains challenging for chemiresistive sensors. Here, chromium-modified double-walled carbon nanotubes (Cr-DWNTs) were prepared using an endohedral filling process and were investigated for preferential xylene detection. X-ray diffraction showed substantial transformation of the CrCl_2_ precursor to rhombohedral Cr_2_O_3_ identified as the dominant crystalline chromium-containing phase. Transmission electron microscopy and elemental analysis supported association of the chromium-containing phase with the DWNT structure. Raman spectroscopy showed retention of the characteristic D, G, and 2D bands with only a small change in the $I_{D}/I_{G}$ ratio, indicating preservation of the graphitic framework after chromium modification. Hall-effect measurements showed positive Hall coefficients for both pristine DWNTs and Cr-DWNTs, confirming hole-dominated transport, while Cr-DWNTs exhibited a lower apparent Hall carrier concentration together with substantially higher carrier mobility and conductivity. Cr-DWNTs exhibited a pronounced preferential response toward xylene over 0.1–10 ppm. The xylene response increased from approximately 0.96% at 0.1 ppm to 5.93% at 10 ppm and was enhanced by approximately 1.9–2.6 times relative to pristine DWNTs. Within 0.1–1 ppm, the low-concentration xylene sensitivity was 2.36% ppm^−1^ ($R^{2}=0.958$), with a calculated limit of detection of approximately 37 ppb. The enhanced response is attributed to the combination of the intrinsic affinity of graphitic carbon toward methyl-substituted aromatics and interfacial electronic transduction within the DWNT network. These results demonstrate that chromium modification provides an effective strategy for amplifying room-temperature xylene selectivity in CNT-based chemiresistive sensors.

## 1. Introduction

Detection of trace levels of volatile organic compounds (VOCs) is very important in environmental monitoring, occupational safety and air-quality assessment [1]. Among them, aromatic hydrocarbons such as benzene, toluene, and xylene, which are generally termed BTX compounds, are of particular concern because they are hazardous, resulting in respiratory irritation and neurological effects at high exposure levels. Even though they are valuable manufacturing ingredients, used in making fuels, paints, coatings and solvent-related activities, their volatility and toxicity requires careful handling and environmental management [2,3,4]. The selective discrimination of individual BTX compounds is still challenging due to their closely related aromatic structures and physicochemical properties that can result in similar or overlapping sensor responses. Xylene is one of the important target analytes among these compounds due to its wide industrial application and the potential for occupational and environmental exposure. Exposure to xylene may cause irritation of the eyes, nose, throat, and respiratory tract, together with neurological effects such as headache, dizziness, drowsiness, fatigue, and impaired coordination. For an 8-h time-weighted average, the United States Occupational Safety and Health Administration (OSHA) has set a permissible exposure limit of 100 ppm for xylene [5], whereas the American Conference of Governmental Industrial Hygienists (ACGIH) recommends a lower threshold limit value of 20 ppm over the same exposure period [6].

Conventional analytical techniques, such as gas chromatography–mass spectrometry, provide reliable separation and identification of xylene and related aromatic VOCs [7]. However, these techniques generally require expensive instrumentation, rigorous application processes and hence it is not ideal for rapid and cost effective testing. This has motivated the development of chemiresistive gas sensors, which have many advantages such as having relatively simple and portable device structure, for low-cost real-time VOC monitoring applications. For a long time metal-oxide semiconductors such as SnO_2_, ZnO, WO_3_, In_2_O_3_, and Fe_2_O_3_ have been widely used as chemiresistive materials for VOC detection because they can provide high responses and relatively short response and recovery times [8,9,10,11]. However, many metal-oxide sensors require elevated operating temperatures to activate adsorbed oxygen species and accelerate surface-reaction and charge-transfer kinetics [12]. High temperature increases power consumption making it complicated to intergrate them into portable and low-power sensing platforms. Although room-temperature operation has been reported, sluggish adsorption, desorption, and reaction kinetics under ambient conditions can result in limited responses or slow and incomplete recovery [13,14,15]. Alternative sensing materials are therefore required to achieve room-temperature operation while maintaining adequate sensitivity, selectivity, response speed, reversibility, and stability.

It is in this light that carbon nanotubes (CNTs) have emerged as promising materials because of their high surface-to-volume ratio, tuneable electrical transport properties, chemical stability, and compatibility with miniaturised devices [16,17]. Adsorption of gas molecules can alter the charge density of individual nanotube or bundles resulting in resistance modulation and hence measurable changes in the resistance of a CNT network. Despite these advantages, pristine CNTs often exhibit limited chemical selectivity because their graphitic surfaces provide relatively nonspecific adsorption and charge-transfer sites. CNT-network responses are further influenced by factors such as the relative proportions of metallic and semiconducting nanotubes, bundling, structural defects, network density, and nanotube–electrode contacts. Several strategies have therefore been investigated to modify CNT sensing behaviour, including covalent and non-covalent functionalisation, metal and metal-oxide decoration, polymer incorporation, heteroatom doping, and endohedral filling [18,19,20,21]. Endohedral filling is particularly interesting because the secondary material is confined within the internal nanotube cavity rather than deposited predominantly on the external graphitic surface. Chimowa et al. reported that filling multi-walled CNTs with vanadium oxide increased their methane response from approximately 0.5% to 1.5%, demonstrating that an internally confined material can tune the electronic and gas-sensing behaviour of a CNT host [22].

Double-walled carbon nanotubes (DWNTs) are particularly attractive hosts for this approach because they have two concentric graphitic walls which combine nanoscale dimensions with greater structural robustness than a single isolated wall [23]. Previous studies have also shown that modification of the outer wall can be achieved while leaving the inner wall comparatively protected, reducing the disruption of electrical transport that may accompany extensive modification of SWNTs. At the same time, the hollow interior provides a confined nanospace for incorporating secondary materials. DWNTs therefore offer a useful platform in which the conductive CNT framework and a confined chemically active phase can be combined. Among potential modifiers, chromium is of particular interest because chromium-containing materials have repeatedly shown enhanced responses towards xylene [10,24]. Cr-doped WO_3_ nanofibers showed enhanced xylene sensing associated with changes in oxygen vacancies and chemisorbed oxygen species [10]; enhanced xylene sensing has also been reported for Cr-doped Co_3_O_4_ [24]. More recently, Cr-doped WO_3_ thin films demonstrated selective xylene detection at room temperature [25]. Collectively, these studies indicate that chromium-containing phases can influence xylene sensing through catalytic, defect-related, interfacial, and electronic effects, providing a rationale for investigating chromium and DWNT hybrid sensors. To achieve endohedral modification of DWNTs, an appropriate chromium precursor that has the right melting point and can be easily oxidized once encapsulated is required. Metal halides have been widely employed as precursors for CNT filling through melt-assisted and related approaches, making chromium halides suitable candidates for introducing chromium-containing species into the confined nanotube structure. CrCl_2_ was selected as the precursor for the endohedral-filling process used in this study. Recent atomic-resolution investigations have demonstrated the formation of single-, double-, and multiple-chain CrCl_2_ structures within CNT cavities, confirming that CrCl_2_ can be integrated easily within the confined nanotube geometry [26]. CNT encapsulation can mitigate environmental degradation by isolating confined material within the nanotube cavity and can additionally restrict particle dimensions and uncontrolled aggregation. Nanotube cavities consequently act as nanoscale reaction vessels in which confined materials can adopt unique structures and compositions different from their corresponding bulk forms [26]. From a sensing perspective, endohedral incorporation also leaves much of the external graphitic surface available for analyte interaction while introducing an internal phase capable of modifying the properties of the CNT host. Encapsulation therefore provides an alternative to external functionalization, where inorganic particles may aggregate, obstruct accessible CNT surfaces, or alter nanotube–nanotube junctions.

Previous works, as outlined earlier, have demonstrated the influence of chromium-containing materials on xylene sensing but none has reported endohedrally Cr-filled DWNTs as chemiresistive sensing materials for VOC detection. CrCl_2_ confinement within CNTs has recently been investigated primarily in the context of low-dimensional structural, electronic, and magnetic properties [26], whereas filled-CNT gas sensors have predominantly employed other encapsulated species and target gases [22,27,28,29]. To the best of our knowledge, a CrCl_2_-derived chromium-containing DWNT hybrid has not previously been investigated for room-temperature xylene sensing. The CrCl_2_ was further oxidized to Cr_2_O_3_ as will be outlined in the methodology. The resulting material was therefore characterized structurally and electrically, and its chemiresistive response toward xylene and competing VOCs was evaluated at room temperature and compared with that of relevant control materials.

## 2. Materials and Methods

### 2.1. Materials

Chromium(II) chloride (CrCl_2_, Sigma-Aldrich, Merck KGaA, Darmstadt, Germany) CAS No. 10049-05-5, 99.99%) was used as the chromium precursor. Xylene, toluene, benzene, methanol, ethanol, acetone, and pentane were used as volatile organic compound (VOC) analytes and were used as received from Sigma-Aldrich. Double-walled carbon nanotubes (DWNTs) were synthesized according to the method reported by Flahaut et al. [30]. All other chemicals and solvents were used as received unless otherwise stated.

### 2.2. Preparation of Cr-DWNT

The chromium-decorated DWNT hybrid, hereafter referred to as Cr-DWNT, was prepared using anhydrous CrCl_2_ as the chromium precursor through a sealed-ampoule melt-filling method. A total of 75 mg of DWNTs was mixed with 1.0 g of anhydrous CrCl_2_. Due to the hygroscopic and air-sensitive nature of CrCl_2_, preparation of the ampoule was carried out in a nitrogen-filled glovebox. The CrCl_2_ precursor and DWNTs were gently ground together using a mortar and pestle to obtain a homogeneous mixture. The resulting mixture was transferred into a quartz ampoule, which was evacuated to approximately 0.1 mbar and sealed under vacuum. The sealed ampoule was then placed in a tubular furnace and heated to 854 °C, approximately 30 °C above the melting point of CrCl_2_. The heating rate was maintained at 5 °C min^−1^, and the sample was held at 854 °C for 24 h. This dwell period was used to ensure complete melting of the CrCl_2_ precursor, with the aim of promoting incorporation of the molten precursor into accessible DWNT cavities by capillary action under vacuum.

Following the dwell period, controlled cooling was carried out in several stages to promote gradual solidification of the chromium-containing phase. The temperature was first reduced from 854 °C to 804 °C at a cooling rate of 0.1 °C min^−1^. The sample was then cooled from 804 °C to 724 °C at a rate of 1 °C min^−1^, followed by cooling from 724 °C to room temperature at approximately 5 °C min^−1^. After cooling to room temperature, the ampoule was opened and the resulting Cr-DWNT material was collected. The product was washed repeatedly with distilled water using a vacuum-filtration setup fitted with a 0.45 μm cellulose nitrate membrane. Washing was continued until the filtrate became colourless, indicating removal of soluble excess chromium-containing species. The washed material was transferred into a pre-weighed sample bottle and frozen for 24 h before being freeze-dried for 48 h to obtain a loose powder and minimise agglomeration.

### 2.3. Sensor Fabrication

**Figure 1:**
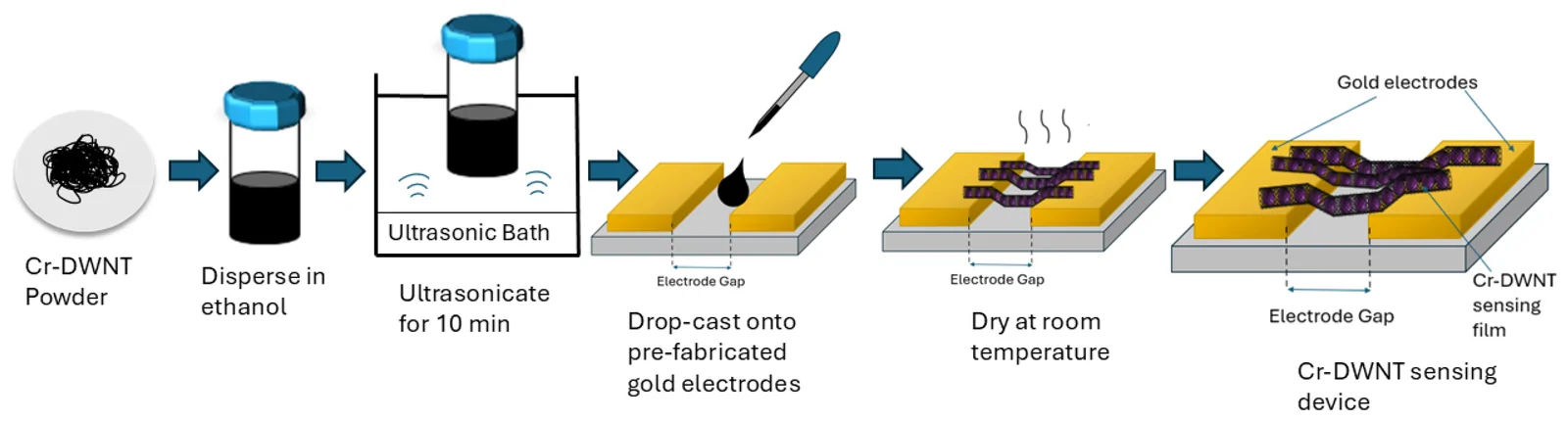
Schematic illustration of the Cr-DWNT sensor-fabrication process.

As illustrated in Figure 1, Cr-DWNT powder (0.5 mg) was dispersed in 0.5 mL of ethanol, corresponding to a dispersion concentration of 1.0 mg mL^−1^. The suspension was sonicated for 10 min in a Branson 1800 ultrasonic bath operating at 40 kHz. A single drop of the dispersion was then drop-cast onto the pre-fabricated gold electrode chips, ensuring that the sensing material covered the electrode gap and formed a continuous conductive pathway between the contacts.. The Pasteur pipette used for deposition was gravimetrically calibrated, with five drops having a total mass of 0.0710 g, corresponding to an average drop volume of approximately 18 μL. This corresponds to a nominal deposited sensing-material mass of approximately 18 μg per device. The coated device were allowed to dry at room temperature for 10 min. The same deposition procedure was used for the pristine DWNT device. The baseline resistance of each device was recorded before gas exposure to confirm electrical continuity of the deposited sensing film.

### 2.4. Gas-Sensing Setup

**Figure 2:**
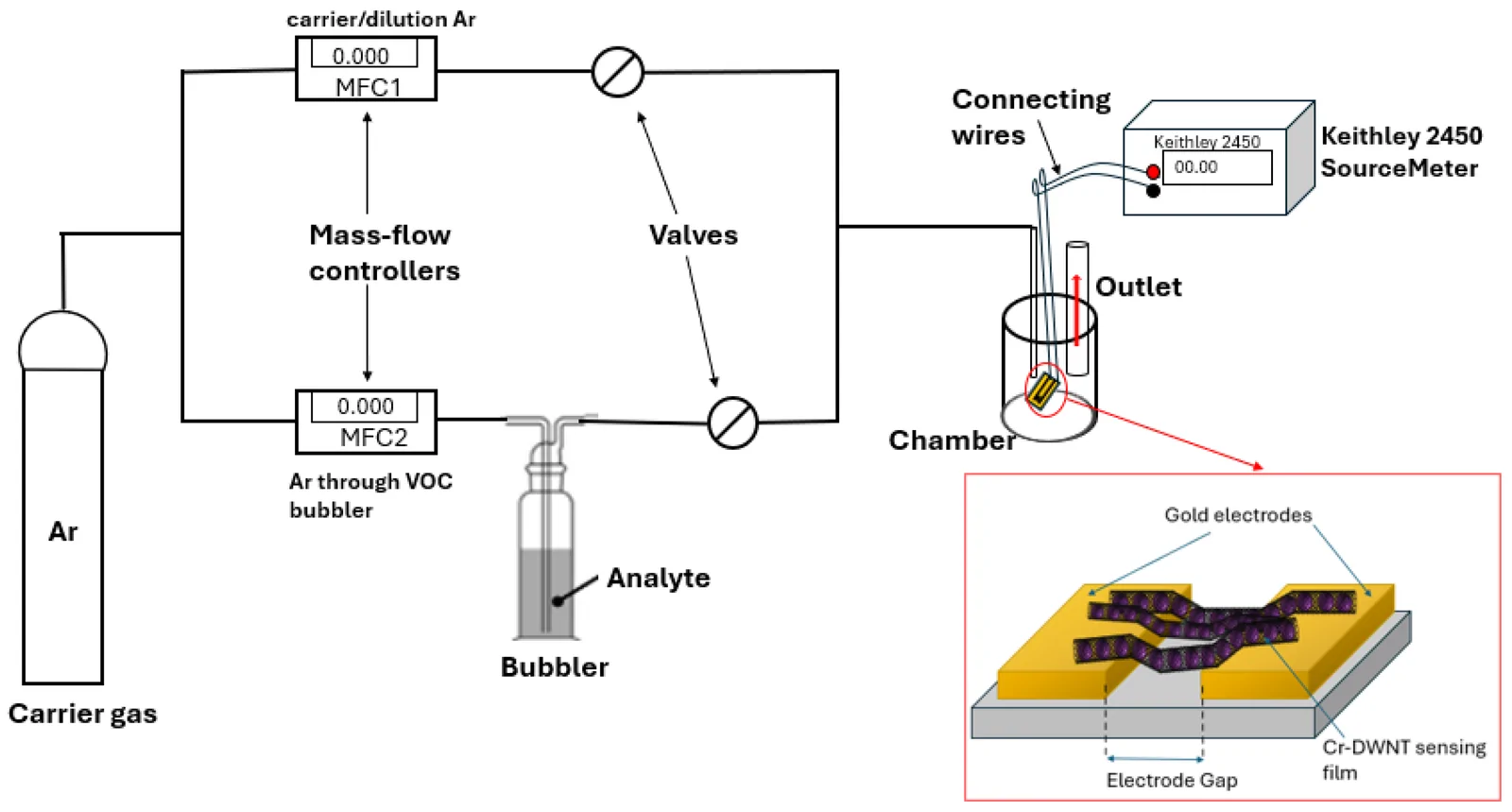
Schematic illustration of the room-temperature gas-sensing system. A custom Python-based program was used to control the mass-flow controllers and Keithley 2450 SourceMeter and to record the electrical response of the sensing device.

Gas-sensing measurements were performed at room temperature using a custom-built dynamic-flow system as shown in Figure 2. Argon was used as an inert carrier and dilution gas. The flow system consisted of two Alicat mass-flow controllers (MFCs), with one MFC controlling the carrier/dilution argon stream and the second controlling the argon stream passing through the liquid VOC bubbler. The MFCs were controlled serially using a Python-based graphical user interface (GUI).

The electrical resistance of the sensing device was monitored continuously using a Keithley 2450 SourceMeter (Keithley Instruments, Cleveland, OH, USA) controlled through the same GUI for automated data acquisition. A constant current of 350 μA was applied during the measurements, with the voltage compliance set to 5.00 V. The VOC vapour stream was generated by bubbling argon through a liquid VOC reservoir maintained at room temperature. The VOC-containing argon stream was subsequently mixed with the carrier/dilution argon stream before entering the sensing chamber. Xylene was used as the target analyte, while toluene, benzene, methanol, ethanol, acetone, and pentane were investigated as interfering VOCs.

For the general VOC-screening experiments, concentrations of 0.1, 1, 5, and 10 ppm were investigated. For detailed concentration-response analysis of Cr-DWNTs toward xylene and toluene, additional concentrations of 0.3, 0.5, and 2 ppm were included, giving a complete concentration series of 0.1, 0.3, 0.5, 1, 2, 5, and 10 ppm. The VOC concentration was controlled by adjusting the relative flow rates of the VOC-containing and carrier/dilution argon streams and was calculated using Equation (1), adapted from Serero et al. [27]:

(1) $$\mathrm{ppm}=\frac{Q_{\mathrm{target}}}{Q_{\mathrm{target}}+Q_{\mathrm{carrier}}}\times \eta \times \frac{10^{\left(A-\frac{B}{C+T}\right)}}{P_{\mathrm{total}}}\times 10^{6}$$

where $Q_{\mathrm{target}}$ is the flow rate of the VOC-containing argon stream from the bubbler and $Q_{\mathrm{carrier}}$ is the flow rate of the carrier/dilution argon stream. Both flow rates are expressed in standard litres per minute (SLPM). The parameter η represents the efficiency coefficient used to account for incomplete transfer of VOC vapour from the liquid phase into the gas stream. The parameters *A*, *B*, and *C* are the Antoine coefficients for the respective VOC, *T* is the temperature in degrees Celsius, and $P_{\mathrm{total}}$ is the total system pressure. The term $10^{\left(A-\frac{B}{C+T}\right)}$ represents the saturated vapour pressure of the analyte. The saturated vapour pressure and $P_{\mathrm{total}}$ were expressed in consistent pressure units, and the factor $10^{6}$ converts the resulting mole fraction to ppm. Water vapour was additionally evaluated as an interferent using the dynamic flow system. Each sensing cycle began with a 5 min argon purge to establish and stabilise the baseline resistance. The VOC-containing gas stream was then introduced into the sensing chamber for 2 min, followed by a 5 min argon purge to remove the analyte and allow sensor recovery. One complete sensing cycle therefore lasted 12 min.

### 2.5. Gas-Sensing Data Analysis

The chemiresistive response was calculated according to:

(2) $$S\left(\%\right)=\frac{R_{g}-R_{a}}{R_{a}}\times 100$$

where $R_{a}$ is the baseline resistance measured in argon immediately before analyte exposure and $R_{g}$ is the resistance measured during exposure to the analyte. The response parameter S(%) was used to compare the sensing behaviour of pristine DWNTs and Cr-DWNTs under comparable experimental conditions. For comparative VOC-response measurements used in the bar-chart analyses, three replicate measurements (n=3) were performed under the same experimental conditions. The reported responses are presented as mean ± standard deviation.

To evaluate the response of Cr-DWNTs under multi-analyte conditions, additional co-exposure measurements were performed using a mixed-interferent stream in the absence and presence of xylene. Measurements were conducted at 0.1, 1, 5, and 10 ppm using the same room-temperature sensing protocol as described above. Each condition was measured in triplicate, and the results are reported as the mean response with the corresponding standard deviation. The mixed-interferent stream contained [ethanol, pentane, methanol, acetone, benzene, toluene and water], while the corresponding xylene-containing mixture was prepared by adding xylene under the same exposure conditions.

The preferential response toward xylene was further evaluated using the selectivity factor:

(3) $$SF=\frac{S_{\mathrm{xylene}}}{S_{\mathrm{interferent}}}$$

where $S_{\mathrm{xylene}}$ is the response toward xylene and $S_{\mathrm{interferent}}$ is the response toward the interfering analyte at the same concentration.

#### 2.5.1. Calibration and Sensitivity

Detailed concentration-response measurements of Cr-DWNTs toward xylene and toluene were performed at 0.1, 0.3, 0.5, 1, 2, 5, and 10 ppm. Measurements were performed in triplicate at each concentration, and the results are reported as mean ± standard deviation. Calibration curves were obtained by plotting the mean sensor response against analyte concentration. Because the response became increasingly non-linear at higher concentrations, the low-concentration region between 0.1 and 1 ppm was analysed separately.

#### 2.5.2. Blank Analysis and Analytical Detection Limits

Blank measurements were performed under the same gas-flow and electrical measurement conditions used for the Cr-DWNT sensing experiments but without introduction of the target analyte. The apparent blank response was calculated using Equation (2).

Short-term baseline noise was determined from the standard deviation of stable baseline regions of the matched blank measurements and was expressed as a relative response percentage. This baseline noise was used as σ for estimation of the analytical detection limits.

The limit of detection (LOD) was estimated according to:

(4) $$LOD=\frac{3\sigma }{m}$$

where σ is the short-term baseline noise obtained from the blank measurements and *m* is the low-concentration xylene sensitivity obtained from the calibration curve.

The limit of quantification (LOQ) was estimated according to:

(5) $$LOQ=\frac{10\sigma }{m}$$

The calculated LOD and LOQ were used as estimates of analytical detection capability and were distinguished from the lowest analyte concentration experimentally investigated.

#### 2.5.3. Response and Recovery Times

The response and recovery kinetics were evaluated at 1 ppm for the investigated VOCs. The response time ($t_{90}$) was defined as the time elapsed between analyte introduction and attainment of 90% of the analyte-induced response. The recovery time was defined as the time elapsed between analyte removal and reversal of 90% of the analyte-induced response. Response and recovery times were determined from the transient sensing curves using the Rise Time/Fall Time analysis in OriginPro 2024 (64-bit) SR1, version 10.1.0.178 (OriginLab Corporation, Northampton, MA, USA). The same analysis criterion was applied to the pristine DWNT and Cr-DWNT to allow direct comparison between the two sensing materials.

### 2.6. Material Characterization

The structural properties of the CrCl_2_ precursor, pristine DWNTs, and Cr-DWNT hybrid were investigated using a Bruker D8 Advance X-ray diffractometer (Bruker AXS GmbH, Karlsruhe, Germany) with Cu Kα radiation (λ=1.5418Å). Diffraction patterns were recorded over a 2θ range of 10–90°. The diffraction patterns were used to evaluate the crystalline phases present in the precursor and recovered Cr-DWNT material. High-resolution transmission electron microscopy (HRTEM) and energy-dispersive X-ray spectroscopy (EDX) were performed using a JEOL JEM-ARM 200F cold field-emission gun (JEOL Ltd., Akishima, Tokyo, Japan), probe Cs-corrected microscope operated at 80 kV. TEM analysis was used to investigate the morphology of the Cr-DWNT material and the spatial distribution of regions of enhanced contrast associated with the chromium-containing phase. EDX was used to determine the elemental composition of the analysed regions, particularly the presence of C, Cr, O, and Cl.

Raman spectroscopy was performed using a Horiba LabRAM HR Evolution Raman spectrometer (HORIBA France SAS, Palaiseau, France) with a 532 nm excitation laser. Spectra were recorded over the Raman-shift range of 500–3000 cm^−1^ for pristine DWNTs and Cr-DWNTs. The characteristic D, G, and 2D bands were analysed to assess changes in the graphitic structure following chromium modification. The degree of defect-related scattering was evaluated using the D-to-G band intensity ratio, $I_{D}/I_{G}$. Electrical transport properties of pristine DWNTs and Cr-DWNTs were evaluated using an ECOPIA HMS-5500 Hall measurement system (Ecopia Corporation, Anyang, Republic of Korea). The Hall coefficient ($R_{H}$), Hall mobility (*μ*_H_), electrical conductivity (σ), and resistivity (ρ) were determined for both materials. The sign of the Hall coefficient was used to assess the dominant carrier type. An apparent Hall carrier concentration was estimated using the single-carrier relationship

(6) $$p_{H}=\frac{1}{qR_{H}},$$

where *q* is the elementary charge and $R_{H}$ is the measured Hall coefficient. Because CNT films consist of interconnected nanotubes and tube–tube junctions rather than a homogeneous bulk semiconductor, $p_{H}$ is treated here as an apparent Hall carrier concentration and is used primarily for comparison between pristine DWNTs and Cr-DWNTs.

## 3. Results

### 3.1. Material Characterization

**Figure 3:**
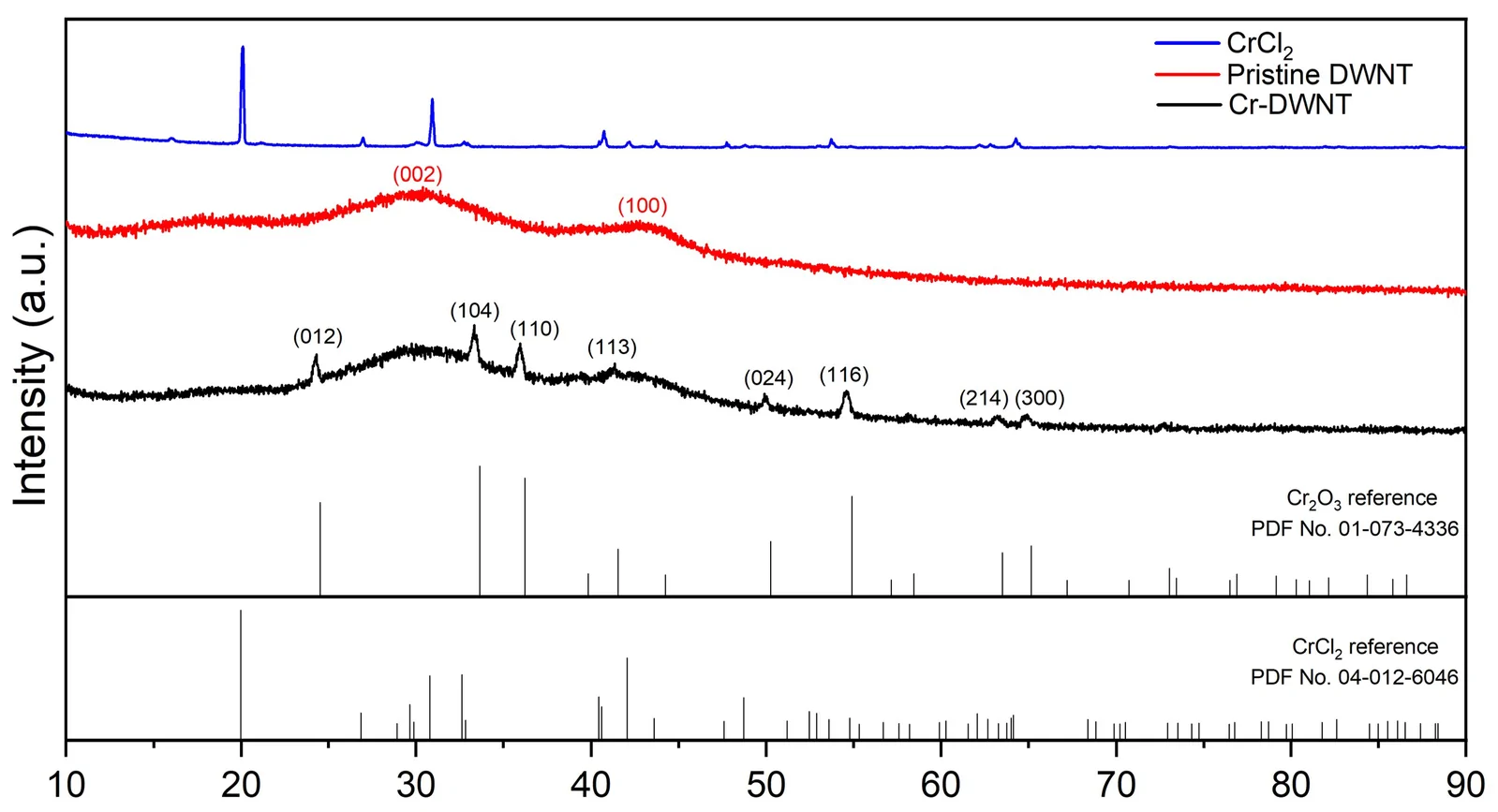
Stacked X-ray diffraction patterns of CrCl_2_, pristine DWNTs, and the Cr-DWNT hybrid, together with reference diffraction patterns for rhombohedral Cr_2_O_3_ (PDF No. 01-073-4336) and crystalline CrCl_2_ (PDF No. 04-012-6046). The experimental patterns are presented in stacked form for clarity. The broad reflections of pristine DWNTs are indexed as the graphitic (002) and (100) planes, while the additional reflections observed for Cr-DWNTs are indexed to the (012), (104), (110), (113), (024), (116), (214), and (300) planes of rhombohedral Cr_2_O_3_.

Figure 3 shows the X-ray diffraction patterns of pristine DWNTs, the CrCl_2_ precursor, and the recovered Cr-DWNT hybrid. Pristine DWNTs exhibited broad diffraction features centred at approximately 2*θ* = 30.19° and 42.67°, which were assigned to the graphitic (002) and (100) reflections [31,32], respectively. In contrast, the CrCl_2_ precursor exhibited several sharp reflections, with the measured pattern showing good agreement with crystalline CrCl_2_ (PDF No. 04-012-6046), including the dominant reflection at approximately $2\theta =20.03^{{}^{\circ}}$.

Following preparation of the Cr-DWNT hybrid, the broad graphitic contribution of the DWNT framework remained visible, while additional crystalline reflections were observed at approximately 24.24°, 33.38°, 35.99°, 41.35°, 49.91°, 54.56°, 63.41°, and 64.85°. These reflections were indexed to the (012), (104), (110), (113), (024), (116), (214), and (300) planes, respectively, of rhombohedral eskolaite Cr_2_O_3_ (PDF No. 01-073-4336). The agreement between the experimental Cr-DWNT diffraction pattern and the Cr_2_O_3_ reference pattern identifies Cr_2_O_3_ as the dominant crystalline chromium-containing phase in the recovered hybrid.

**Figure 4:**
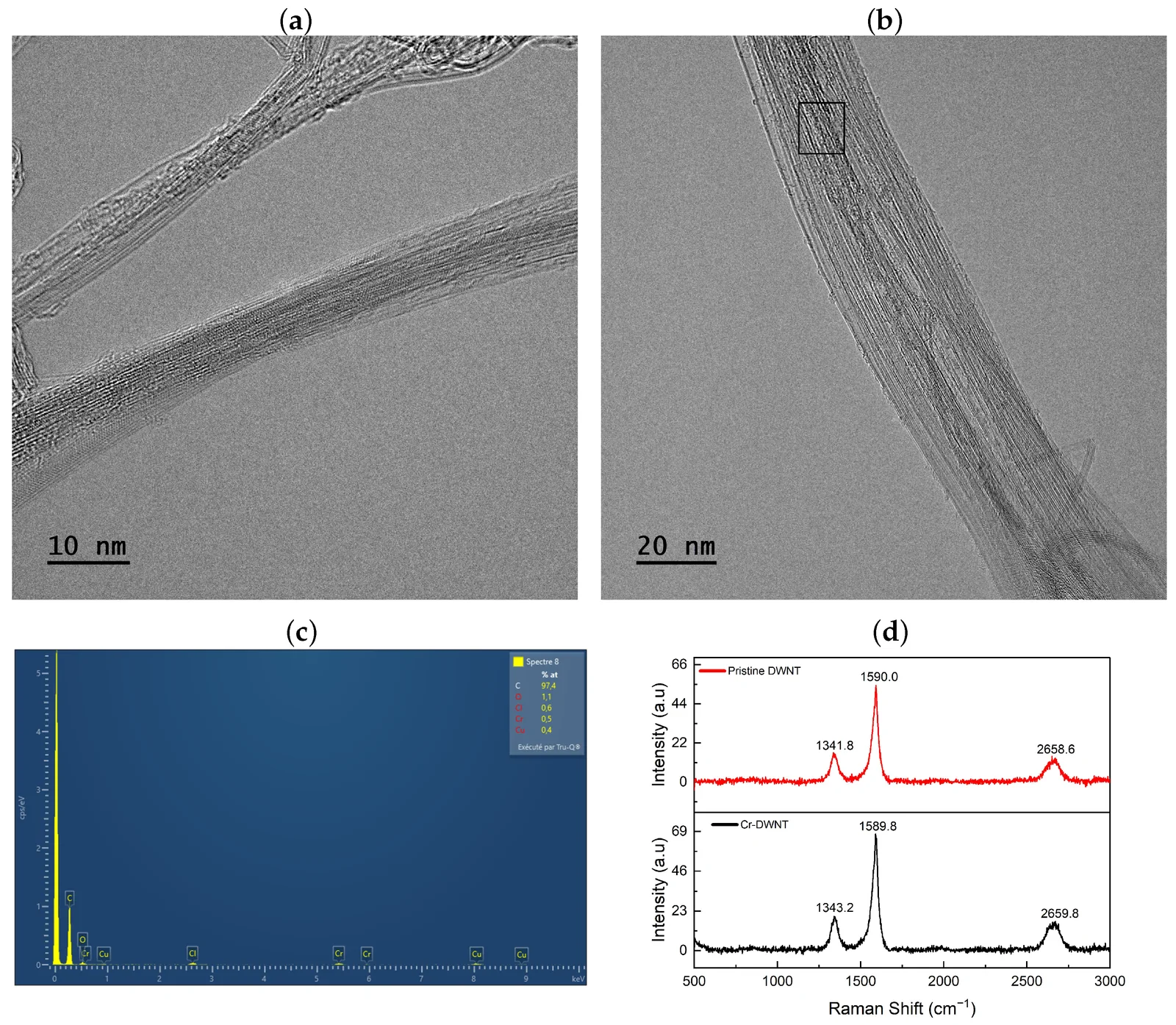
HRTEM, EDX, and Raman characterization of the Cr-DWNT hybrid. (**a**,**b**) HRTEM images showing the retained tubular morphology of the DWNT framework and regions of enhanced electron contrast associated with the nanotube structure. The scale bars correspond to 10 nm in (**a**) and 20 nm in (**b**). (**c**) Representative EDX spectrum acquired from the boxed region indicated in (**b**). (**d**) Raman spectra of the pristine DWNT and Cr-DWNT showing the characteristic D, G, and 2D bands.

The morphology and elemental composition of the Cr-DWNT hybrid were further examined by HRTEM and EDX. As shown in Figure 4a,b, the tubular morphology of the DWNT framework was retained following chromium modification. Regions of enhanced electron contrast extending along the nanotube axis were also observed within the nanotube boundaries. However, the available HRTEM images did not resolve lattice fringes sufficiently to permit independent crystallographic identification of the chromium-containing phase. A representative HRTEM image of pristine DWNTs is provided in Figure S2 for comparison with the morphology of Cr-DWNT. The EDX spectrum acquired from the region indicated in Figure 4b is shown in Figure 4c. The analysed region was predominantly carbon, with C, O, Cl, and Cr accounting for 97.4, 1.1, 0.6, and 0.5 at.%, respectively. A minor Cu contribution of approximately 0.4 at.% was also detected and originated from the TEM support grid.

Raman spectroscopy was used to evaluate changes in the graphitic DWNT framework following chromium modification. As shown in Figure 4d, pristine DWNTs exhibited characteristic D and G bands at approximately 1341.8 and 1590.0 cm^−1^, respectively, with an $I_{D}/I_{G}$ ratio of 0.324. Following chromium modification, the D and G bands were observed at approximately 1343.2 and 1589.8 cm^−1^, corresponding to shifts of approximately +1.4 and −0.2 cm^−1^, respectively. The $I_{D}/I_{G}$ ratio changed slightly from 0.324 for pristine DWNTs to 0.309 for Cr-DWNTs. The characteristic 2D band was retained in both materials, occurring at approximately 2658.6 cm^−1^ for pristine DWNTs and 2659.8 cm^−1^ for Cr-DWNTs.

### 3.2. Electrical Transport Properties

The room-temperature electrical transport properties of pristine DWNTs and Cr-DWNTs were evaluated by Hall-effect measurements and are summarized in Table 1. Both pristine DWNTs and Cr-DWNTs exhibited positive Hall coefficients, with values of 4.2533 and 7.9046 cm^3^ C^−1^, respectively, indicating hole-dominated transport in both materials. The corresponding apparent Hall carrier concentration decreased from approximately $1.47\times 10^{18}$ cm^−3^ for pristine DWNTs to $7.90\times 10^{17}$ cm^−3^ for Cr-DWNTs. In contrast, the Hall mobility increased from approximately $2.64\times 10^{2}$ to $1.17\times 10^{3}$ cm^2^ V^−1^ s^−1^, while the conductivity increased from approximately 62.1 to 147.9 S cm^−1^. The corresponding resistivity decreased from $1.61\times 10^{-2}$ to $6.76\times 10^{-3}$ Ω cm.

### 3.3. Gas-Sensing Performance

**Figure 5:**
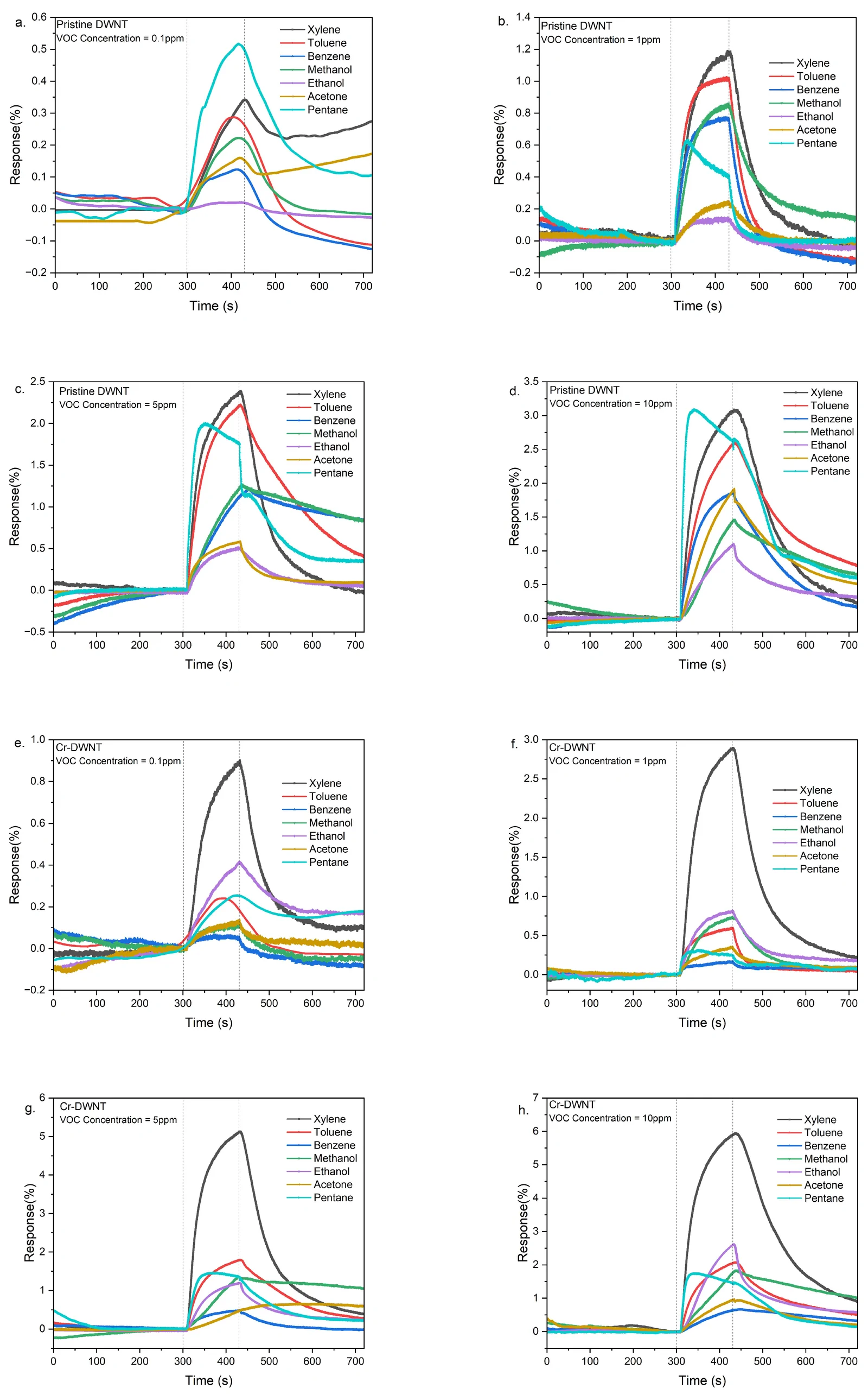
Room-temperature transient gas-sensing responses of (**a**–**d**) pristine DWNTs and (**e**–**h**) Cr-DWNTs toward xylene, toluene, benzene, methanol, ethanol, acetone, and pentane at concentrations ranging from 0.1–10 ppm. The dashed lines indicate the analyte exposure period.

Following structural and electrical characterization, the room-temperature gas-sensing behaviour of pristine DWNTs and Cr-DWNTs was evaluated toward xylene, toluene, benzene, methanol, ethanol, acetone, and pentane over the concentration range of 0.1–10 ppm. The corresponding response profiles are shown in Figure 5. Across the xylene measurements, the mean baseline resistance, $R_{a}$, was approximately 12.74±0.49Ω for pristine DWNTs and 29.60±0.35Ω for Cr-DWNTs. Pristine DWNTs (Figure 5a–d) exhibited measurable responses toward all investigated VOCs, with the response generally increasing with analyte concentration. Exposure to the VOCs predominantly produced an increase in resistance, followed by a decrease toward the baseline after analyte removal. Several analytes produced responses of comparable magnitude. Among the aromatic VOCs, pristine DWNTs exhibited the general response order xylene > toluene > benzene. At 1 ppm, the responses toward xylene and toluene were approximately 1.18 and 1.03%, respectively, while at 5 ppm the corresponding responses were approximately 2.40 and 2.22%. Pentane also produced relatively high responses at some concentrations (see Figure S1 for the bar graph summaries).

Cr-DWNTs (Figure 5e–h) exhibited a different response distribution, with xylene producing the highest response at each investigated concentration. The xylene response increased from approximately 0.90% at 0.1 ppm to 2.88, 5.10, and 5.91% at 1, 5, and 10 ppm, respectively. At 1 ppm, the response toward xylene was approximately 2.88%, compared with approximately 0.80% for ethanol, 0.72% for methanol, and 0.58% for toluene, while benzene, acetone, and pentane produced smaller responses. Relative to pristine DWNTs, the xylene response of Cr-DWNTs increased by approximately 2.6-, 2.4-, 2.1-, and 1.9-fold at 0.1, 1, 5, and 10 ppm, respectively. The incremental increase in xylene response became smaller at the higher concentrations, particularly between 5 and 10 ppm, indicating increasingly sub-linear concentration-response behaviour.

**Figure 6:**
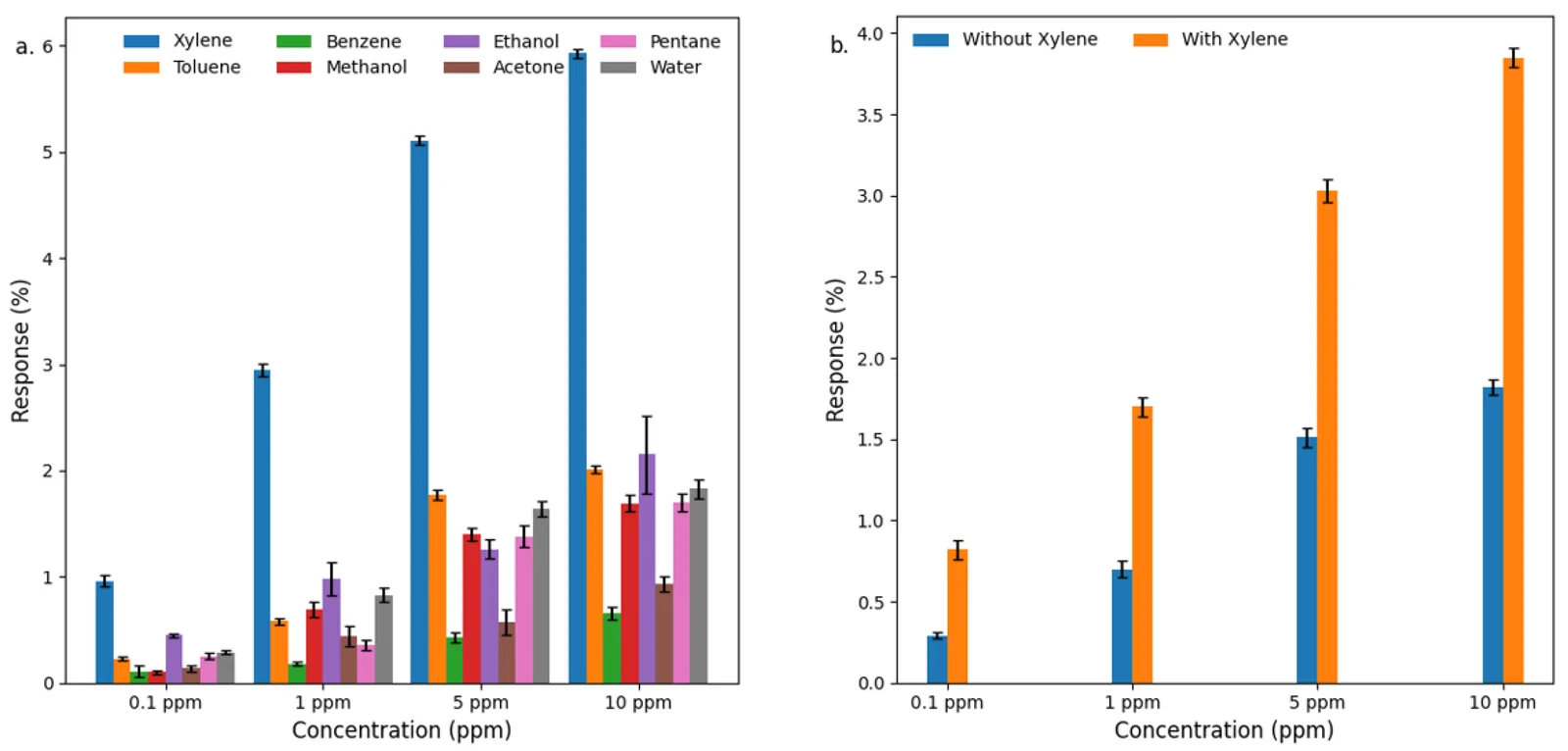
(**a**) Room-temperature response of Cr-DWNTs toward xylene, toluene, benzene, methanol, ethanol, acetone, pentane, and water vapour over the investigated concentration range of 0.1–10 ppm. (**b**) Response of Cr-DWNTs to the mixed-interferent stream in the absence and presence of xylene over the same concentration range. Bars represent the mean response of three replicate measurements (n=3), and error bars represent the corresponding standard deviation.

The response profiles also differed among the investigated analytes. Pentane reached its maximum response considerably earlier than xylene, whereas the xylene response increased more gradually during the exposure period. These kinetic differences were subsequently quantified using the $t_{90}$ response and recovery times. To compare the response magnitudes across the investigated analytes more directly, the Cr-DWNT responses were summarized together with water as an additional interferent in Figure 6a. Xylene produced the highest response at each investigated concentration. At 0.1 ppm, the xylene response was approximately 0.90%, compared with 0.42% for ethanol, 0.31% for water, 0.26% for pentane, and 0.24% for toluene. At 1 ppm, the xylene response increased to approximately 2.88%, compared with 0.84% for water, 0.80% for ethanol, 0.72% for methanol, and 0.58% for toluene. Xylene remained the highest-response analyte at 5 and 10 ppm, although ethanol was the strongest organic interferent at the highest investigated concentration. Across the investigated concentration range, the xylene response was approximately 2.9–3.4 times greater than the response toward water.

The preferential response toward xylene was further quantified using the selectivity factor defined in Equation (3). The calculated selectivity factors were greater than unity for all investigated interferents and concentrations, ranging from 2.13 to 16.39 (Table S1). Within the BTX group, the xylene-to-toluene selectivity factor ranged from 2.87 to 5.09, while the xylene-to-benzene selectivity factor ranged from 8.73 to 16.39. The selectivity factor relative to water remained between 3.12 and 3.55 across the investigated concentration range. The complete selectivity-factor values are provided in Table S1. To further evaluate the behaviour of Cr-DWNTs under multi-analyte conditions, the sensor was exposed to a mixed-interferent stream in the absence (without xylene) and presence of xylene (with xylene) (Figure 6b). The mixed interferents produced a measurable response that increased with concentration. However, addition of xylene produced a clear further increase in the sensor response at each investigated concentration. The difference between the responses obtained with and without xylene also increased with concentration, demonstrating that a distinguishable xylene-associated response was retained in the presence of competing VOCs. These results indicate that, although coexisting analytes contribute to the total chemiresistive response, they do not completely suppress the preferential response of Cr-DWNTs toward xylene.

**Figure 7:**
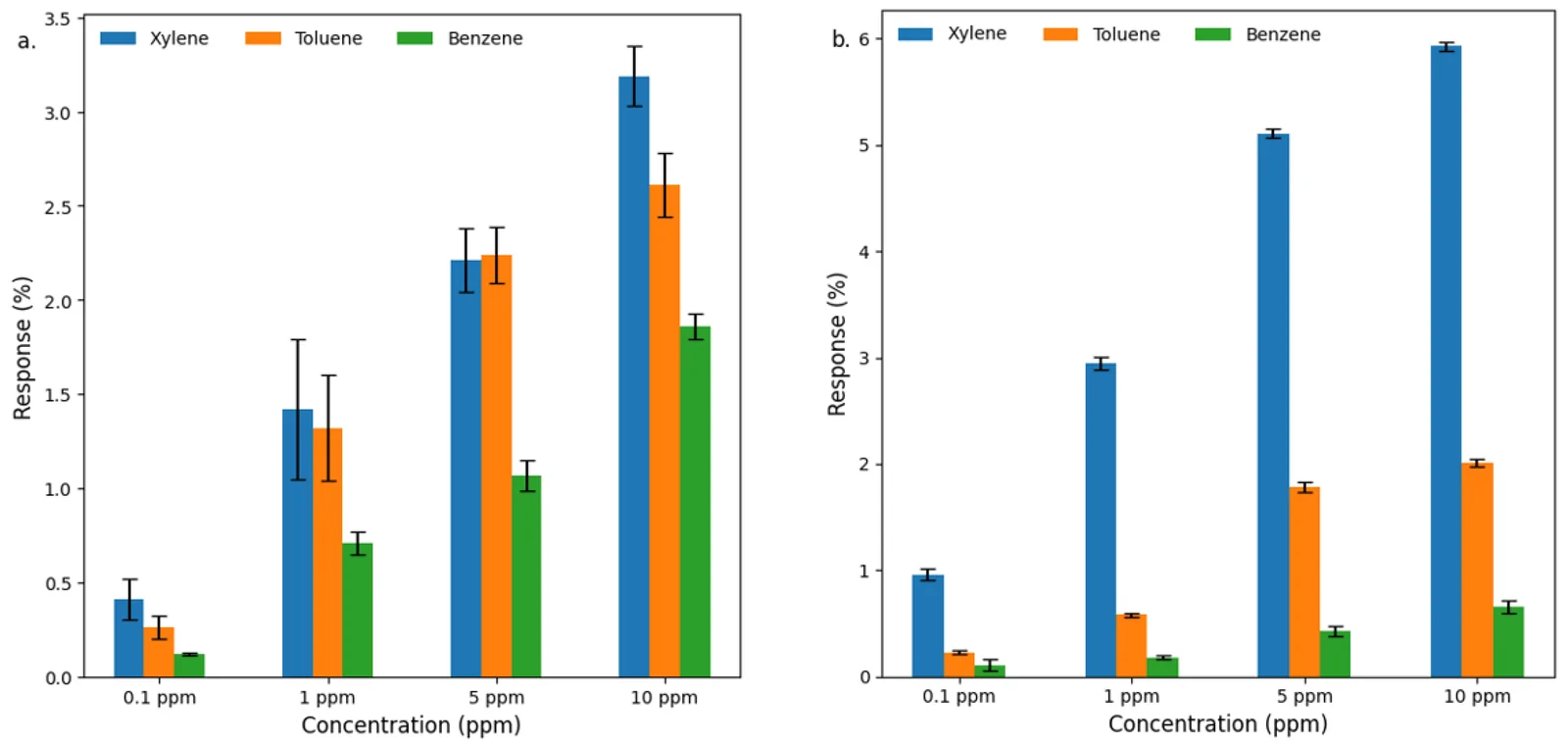
Room-temperature BTX responses of (**a**) pristine DWNTs and (**b**) Cr-DWNTs toward xylene, toluene, and benzene over the investigated concentration range of 0.1–10 ppm. Bars represent the mean response of three replicate measurements (n=3), and error bars represent the corresponding standard deviation.

Because benzene, toluene, and xylene are structurally closely related, their responses were examined separately to assess discrimination within the BTX group (Figure 7). Pristine DWNT (Figure 7a) exhibited the response order xylene > toluene > benzene, although the responses toward xylene and toluene remained relatively close across the investigated concentration range. Cr-DWNTs (Figure 7b) retained the same response order but exhibited greater separation between xylene and the other aromatic VOCs. The xylene-to-toluene response ratios were approximately 3.7, 4.8, 2.9, and 2.9 at 0.1, 1, 5, and 10 ppm, respectively. At 1 ppm, Cr-DWNT produced responses of approximately 2.88, 0.58, and 0.18% toward xylene, toluene, and benzene, respectively, compared with approximately 1.18, 1.03, and 0.78% for pristine DWNT.

In addition to response magnitude, the sensing profiles showed differences in response and recovery behaviour among the investigated VOCs. The corresponding $t_{90}$ response and recovery times at 1 ppm are summarized in Table 2.

Table 2 summarizes the $t_{90}$ response and recovery times of pristine DWNTs and Cr-DWNTs toward the investigated VOCs at 1 ppm. For most analytes, Cr-DWNTs exhibited longer response times than pristine DWNTs. For xylene, the response time increased from 57.4 s for pristine DWNTs to 63.1 s for Cr-DWNTs, while the corresponding recovery time increased from 121.8 to 135.1 s. Xylene and toluene exhibited similar response times on Cr-DWNTs, with values of 63.1 and 64.8 s, respectively. Pentane showed the shortest response time for both materials, at approximately 9 s, although its recovery time differed substantially between pristine DWNTs and Cr-DWNTs, increasing from 35.4 to 180.0 s. For xylene, both the response and recovery times remained within the 120 s exposure and 300 s purge periods used during the sensing measurements.

#### Concentration-Response Behaviour and Analytical Performance

**Figure 8:**
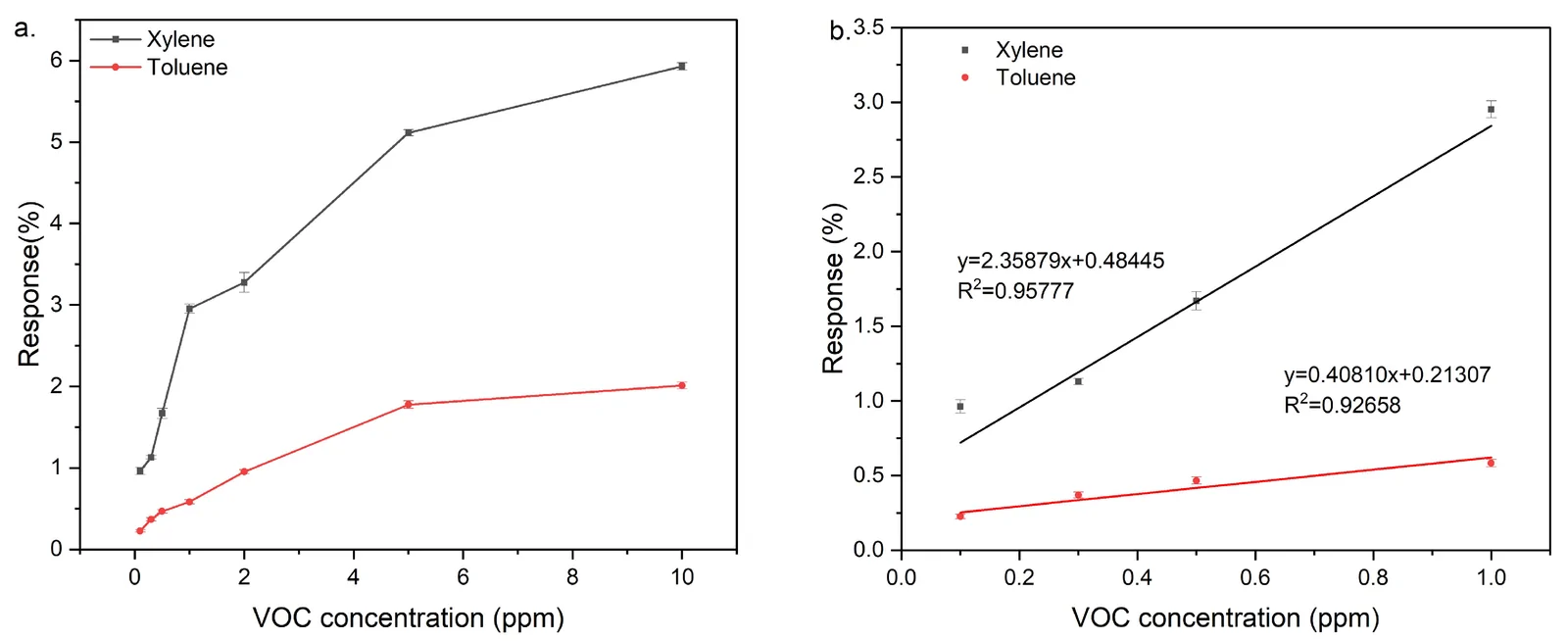
Concentration-dependent room-temperature responses of Cr-DWNTs toward xylene and toluene. (**a**) Response over the complete investigated concentration range of 0.1–10 ppm. (**b**) Linear regression within the low-concentration range of 0.1–1 ppm. Error bars represent the standard deviation of three measurements (n=3).

To further quantify the concentration-dependent behaviour of Cr-DWNTs, detailed measurements were performed toward xylene and toluene over the range of 0.1–10 ppm. The resulting calibration curves are shown in Figure 8.

The responses toward both analytes increased with increasing concentration, while the xylene response remained higher throughout the investigated range. The mean xylene response increased from approximately 0.96% at 0.1 ppm to 5.93% at 10 ppm, whereas the corresponding toluene response increased from approximately 0.24% to 2.05%. At higher concentrations, particularly between 5 and 10 ppm, the incremental increase in response became smaller for both analytes, indicating increasingly sub-linear concentration-response behaviour. To evaluate the analytical sensitivity in the low-concentration region, linear regression was performed over 0.1–1 ppm. The xylene calibration yielded a sensitivity of approximately 2.36% ppm^−1^ with $R^{2}=0.958$, whereas the corresponding toluene sensitivity was approximately 0.408% ppm^−1^ with $R^{2}=0.927$. The xylene response gradient was therefore approximately 5.78 times greater than that of toluene.

Three matched blank measurements produced an average apparent blank response of approximately 0.015%, while the mean response toward 0.1 ppm xylene was approximately 0.963%. The corresponding short-term baseline noise was 0.0293%. Using the low-concentration xylene sensitivity of 2.36% ppm^−1^ and the measured baseline noise, the calculated limit of detection (LOD), according to Equation (4), was approximately 0.037 ppm (37 ppb). The corresponding limit of quantification (LOQ), calculated according to Equation (5), was approximately 0.124 ppm (124 ppb). These values represent calculated analytical limits, whereas 0.1 ppm (100 ppb) was the lowest xylene concentration experimentally investigated.

## 4. Discussion

### 4.1. Structural Consequences of Chromium Modification

Comparison of the starting CrCl_2_ precursor with the recovered Cr-DWNT material indicates substantial transformation of the crystalline chromium-containing phase. The characteristic CrCl_2_ diffraction fingerprint was no longer the dominant inorganic contribution in the Cr-DWNT, whereas reflections corresponding to rhombohedral Cr_2_O_3_ were observed. XRD therefore identifies Cr_2_O_3_ as the dominant crystalline chromium-containing phase in the recovered material. Although the disappearance of the CrCl_2_ fingerprint and emergence of Cr_2_O_3_ reflections demonstrate substantial transformation during preparation and recovery of the hybrid, the diffraction data do not resolve the specific stage at which oxidation occurred. The TEM observations provide complementary information on the spatial association of the chromium-containing material with the DWNT framework. Regions of enhanced electron contrast extending along the nanotube axis and within the nanotube boundaries are consistent with partial endohedral incorporation of a chromium-containing phase. Similar elongated confined structures have been reported for CrCl_2_ chains within CNTs [26] and for other inorganic species incorporated within DWNTs [27]. Nevertheless, the available HRTEM images do not resolve lattice fringes sufficiently to permit independent crystallographic identification of the confined material. Consequently, the assignment of Cr_2_O_3_ as the dominant crystalline chromium-containing phase is based primarily on XRD, while TEM provides morphological evidence for association of chromium-containing material with the DWNT architecture.

The EDX results provide further compositional support for this interpretation. The detection of Cr and O is consistent with the Cr_2_O_3_-dominated phase identified by XRD, whereas the residual Cl signal indicates that complete transformation of the original CrCl_2_ precursor cannot be assumed. Taken together, the XRD, TEM, and EDX results therefore support the formation of a Cr_2_O_3_-dominated chromium-containing DWNT hybrid with a minor residual chlorine contribution. Raman spectroscopy further shows that chromium modification and the associated high-temperature treatment did not substantially disrupt the graphitic DWNT framework. Only minor shifts were observed in the D and G bands, while the $I_{D}/I_{G}$ ratio decreased slightly from 0.324 to 0.309 rather than increasing. The retained 2D band provides additional evidence that the graphitic character of the DWNT framework was preserved after processing. The comparatively subtle Raman changes may also reflect the low proportion of chromium-containing material detected by EDX, since carbon remained the dominant component of the analysed region. Thus, chromium modification produced a structurally distinct hybrid while preserving the predominantly graphitic character of the DWNT network.

### 4.2. Preferential Xylene Response and BTX Discrimination

Pristine DWNTs exhibited relatively broad responses toward the investigated VOCs, with several analytes producing responses of comparable magnitude. Such behaviour is characteristic of CNT-based chemiresistors, in which adsorbed molecules can perturb electrical transport through charge transfer, molecular polarization, and modulation of CNT–CNT junction resistance [33]. This broad response distribution resulted in relatively limited discrimination between several of the investigated analytes. Within the aromatic VOC group, pristine DWNTs exhibited the response order xylene > toluene > benzene. This trend is consistent with previous calculations showing that increasing methyl substitution strengthens the interaction of methylbenzenes with graphitic carbon [34]. However, the relatively small separation between the xylene and toluene responses indicates that the pristine DWNT alone provides only limited discrimination between these closely related aromatic compounds.

Chromium modification substantially altered this response distribution. Cr-DWNTs retained the same BTX response hierarchy, xylene > toluene > benzene, but the separation between xylene and the other aromatic VOCs increased markedly. The chromium-containing phase therefore does not appear to generate a new intrinsic BTX preference. Instead, it amplifies the difference between responses that is already present in the graphitic DWNT host. The sensing behaviour is consequently more appropriately described as enhanced xylene discrimination within the BTX group rather than absolute xylene selectivity. This interpretation is further supported by the comparison with the complete VOC panel. The xylene response was enhanced relative to pristine DWNTs throughout the investigated concentration range, whereas comparable enhancement was not observed uniformly for the other analytes. Chromium modification therefore does not produce a nonspecific amplification of all vapours; rather, the magnitude of the chemiresistive perturbation remains strongly dependent on analyte identity.

Water also produced a concentration-dependent response, consistent with the known sensitivity of CNT-based sensing networks to adsorbed water [35,36]. Nevertheless, the xylene response remained approximately 2.9–3.4 times greater than the response toward water over the investigated concentration range. This observation should not be interpreted as evidence of humidity independence, since water was evaluated as an individual interferent rather than as a controlled background relative humidity. Evaluation under defined humidity conditions will therefore be required to determine the practical humidity tolerance of the sensor. Toluene provides a particularly relevant comparison because it is structurally similar to xylene. Both molecules contain an aromatic π-conjugated framework, but xylene contains two methyl substituents whereas toluene contains one. The persistence of a larger xylene response throughout the investigated concentration range, together with the approximately 5.78-fold greater low-concentration response-gradient, shows that Cr-DWNTs can distinguish between these closely related aromatic compounds rather than responding uniformly to BTX vapours. The co-exposure measurements further support the preferential response of Cr-DWNTs toward xylene under more complex gas conditions. Although the interferent mixture itself produced a measurable response, the addition of xylene resulted in a further increase at every investigated concentration. This indicates that the xylene-associated response was retained in the presence of competing VOCs rather than being completely masked by the interferent background. The co-exposure measurements further support the preferential response of Cr-DWNTs toward xylene under more complex gas conditions. Although the interferent mixture itself produced a measurable response, the addition of xylene resulted in a further increase at every investigated concentration. This indicates that the xylene-associated response was retained in the presence of competing VOCs rather than being completely masked by the interferent background. Together with the selectivity-factor analysis, these results demonstrate that the preferential xylene response of Cr-DWNT is retained even when competing VOCs are simultaneously present.

### 4.3. Concentration-Response Behaviour, Sensing Kinetics, and Analytical Performance

The concentration-dependent measurements provide further insight into the sensing behaviour of Cr-DWNTs. Although the responses toward both xylene and toluene increased with increasing concentration, the incremental response became smaller at higher concentrations, particularly between 5 and 10 ppm. This increasingly sub-linear behaviour may reflect progressive occupation of adsorption sites or electrically active interaction sites within the sensing network, such that additional analyte molecules produce progressively smaller changes in the measured resistance. The principal effect of chromium modification is therefore an increase in response magnitude and analyte discrimination rather than an improvement in response or recovery speed. Nevertheless, the xylene response and recovery remained within the exposure and purge periods used during the measurements.

The low-concentration calibration further demonstrated a substantially greater response gradient toward xylene than toward toluene. Together with the persistent separation between the two concentration-response curves, this supports preferential xylene sensing in the low-concentration regime. The calculated LOD of approximately 37 ppb and LOQ of approximately 124 ppb indicate the potential analytical capability of the Cr-DWNT sensor at sub-ppm xylene concentrations. However, these values are calculated limits derived from the measured baseline noise and calibration slope. Because 0.1 ppm was the lowest xylene concentration experimentally investigated, the 37 ppb LOD should be regarded as an estimated analytical limit rather than an experimentally validated detection concentration. The sensing performance may also be influenced by the amount of material deposited on the electrodes and the concentration of the sensing dispersion. In the present study, these parameters were kept constant and were not systematically optimized. Future optimization of the dispersion concentration and deposited mass may therefore provide an additional route for improving sensor response and reproducibility.

### 4.4. Proposed Gas-Sensing Mechanism

The sensing trends discussed above indicate that chromium modification enhances the electrical response toward xylene without creating a new BTX response hierarchy. The preferential xylene response of Cr-DWNTs is therefore proposed to arise from a coupled adsorption–electronic transduction mechanism involving two complementary contributions: the intrinsic adsorption characteristics of the graphitic DWNT framework and electronic modification of the nanotube network by the Cr_2_O_3_-dominated chromium-containing phase.

The first contribution originates from the interaction of BTX molecules with the graphitic DWNT framework. The aromatic π system of benzene, toluene, and xylene can interact with the delocalized π-electron system of graphitic carbon through dispersion and electrostatic interactions. Increasing methyl substitution increases molecular polarizability and can strengthen dispersive interactions with the graphitic surface. Molecular simulations of benzene, toluene, and *p*-xylene adsorption on graphite at 298 K reported the affinity order xylene > toluene > benzene, with zero-loading isosteric heats of adsorption of approximately 36, 43, and 51 kJ mol^−1^, respectively [37]. This order is consistent with the response hierarchy observed for pristine DWNTs in the present study. Chromium modification therefore does not appear to generate an entirely new BTX preference, but instead amplifies a molecular preference already present in the graphitic DWNT host.

The second contribution arises from electronic modification of the DWNT network by the chromium-containing phase. Structural characterization identified rhombohedral Cr_2_O_3_ as the dominant crystalline chromium-containing phase in the recovered Cr-DWNT material, while TEM and EDX supported association of chromium-containing material with the DWNT architecture. Raman spectroscopy further showed that the predominantly graphitic structure of the DWNT framework was retained following chromium modification. The proposed sensing mechanism is therefore considered in terms of molecular adsorption on the graphitic DWNT framework coupled to electronic modification of the conducting nanotube network by the Cr_2_O_3_-dominated chromium-containing phase, rather than by the original CrCl_2_ precursor.

Cr_2_O_3_ is a wide-band-gap p-type semiconductor, with reported optical band-gap values of approximately 3.1–3.4 eV [38]. Given the substantially greater conductivity of the DWNT network, the Cr_2_O_3_ phase is unlikely to constitute the principal current-carrying pathway through the hybrid. Instead, intimate contact between Cr_2_O_3_ and DWNTs is proposed to modify the local carrier distribution and electrostatic environment of the surrounding nanotube network. When Cr_2_O_3_ and DWNTs are brought into electronic contact, equilibration of their electronic chemical potentials may result in interfacial charge redistribution. CNT work functions have been reported in the approximate range of 4.7–4.9 eV [39], while values close to 5.0–5.1 eV have been reported for some p-type Cr_2_O_3_ films [38]. However, these values depend strongly on factors including surface termination, stoichiometry, defects, adsorbates, nanotube chirality, and processing history. The exact direction and magnitude of equilibrium charge transfer across the Cr_2_O_3_–DWNT interface therefore cannot be determined from the literature work-function values alone.

The Hall-effect measurements nevertheless provide direct experimental evidence that chromium modification changes the transport state of the DWNT network. Both pristine DWNTs and Cr-DWNTs remained hole-dominated following chromium modification, showing that incorporation of the chromium-containing phase did not reverse the majority-carrier polarity of the nanotube network. However, Cr-DWNTs exhibited an apparent Hall hole concentration approximately 46% lower than that of pristine DWNTs, while the Hall mobility increased by approximately 4.4-fold and the conductivity also increased. These changes demonstrate that the chromium-containing phase modifies the baseline electrical transport state of the DWNT network. The Hall-derived carrier concentrations should be interpreted as apparent rather than absolute microscopic carrier densities because electrical transport through CNT films occurs through heterogeneous networks containing metallic and semiconducting nanotubes, inter-tube junctions, and electrode contacts. Hall measurements of CNT films can provide reliable information on dominant carrier polarity, whereas carrier concentrations obtained using a single-carrier approximation may differ from the microscopic carrier density [40]. Nevertheless, the consistent changes in apparent carrier concentration, Hall mobility, and conductivity provide a physical basis for considering electronic sensitization of the DWNT network as part of the enhanced chemiresistive response.

For an ideal p-type conductor,

(7) σ=qpμ,

where *p* is the hole concentration and *μ* is the carrier mobility. For small perturbations,

(8) $$\frac{\Delta R}{R}\approx -\frac{\Delta p}{p}-\frac{\Delta \mu }{\mu }.$$

Although Equation (8) provides only a qualitative description for a percolating CNT film, in which CNT–CNT and electrode–CNT junctions also contribute to the measured resistance, it illustrates an important consequence of the Hall results. A lower initial effective hole concentration can increase the fractional electrical effect of a given adsorption-induced change in carrier population. Thus, for a comparable absolute perturbation Δp, the relative change |Δp|/p becomes larger when the initial effective hole concentration is lower. A related carrier-concentration sensitization effect has been reported for Cr-doped p-type NiO, where Cr incorporation reduced the hole concentration and enhanced the chemiresistive response toward xylene and toluene [41]. The lower apparent Hall hole concentration of Cr-DWNTs therefore provides a physically plausible contribution to its enhanced fractional xylene response.

**Figure 9:**
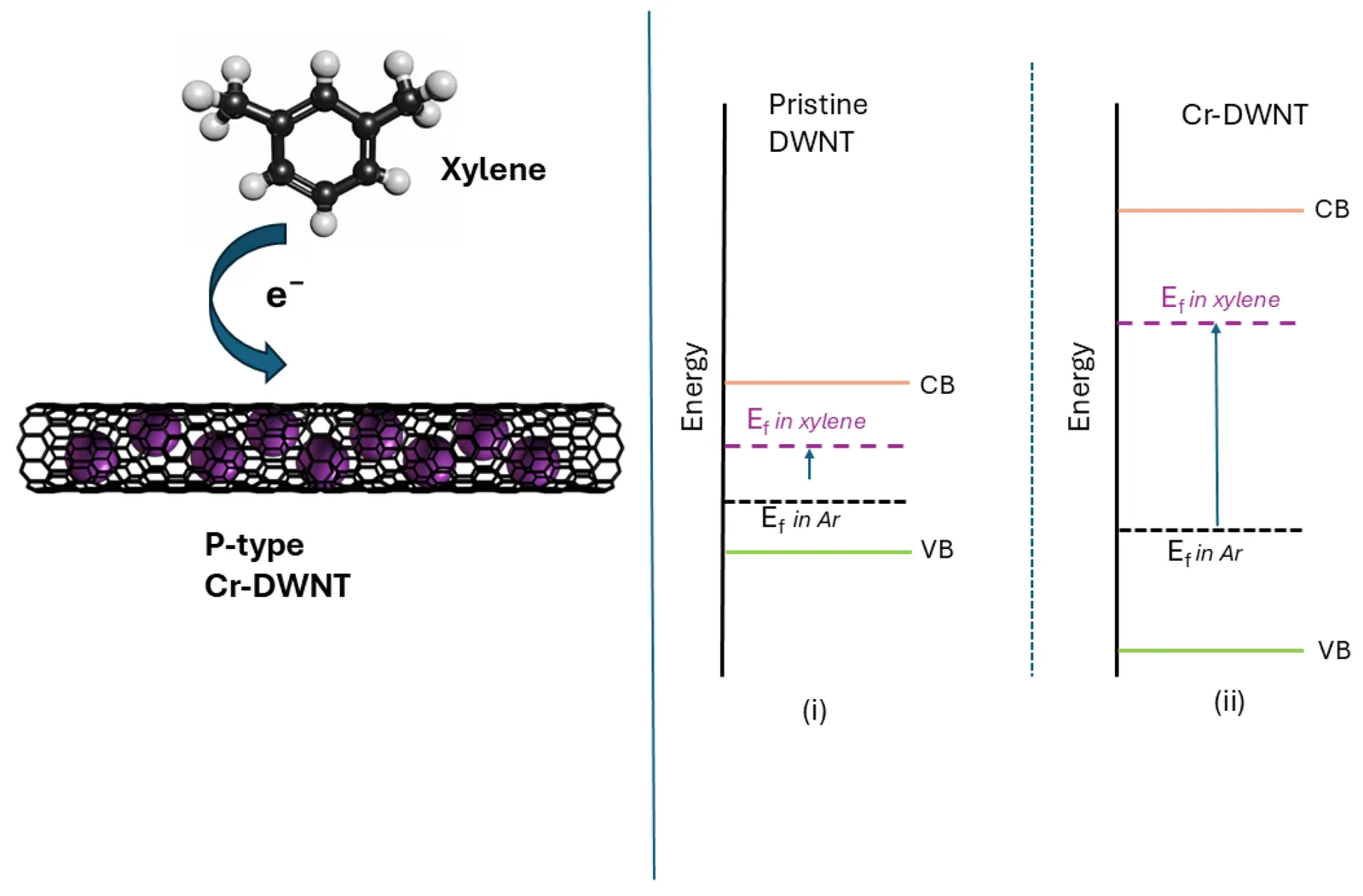
Proposed Fermi-level modulation during xylene sensing by pristine DWNTs and Cr-DWNTs. The schematic on the left illustrates the proposed electron donation from xylene to the p-type Cr-DWNT sensing network. The band diagrams on the right represent (**i**) pristine DWNT and (**ii**) Cr-DWNT. CB and VB denote the conduction and valence bands, respectively. The black dashed lines indicate the Fermi level in Argon ($E_{f}$* in Ar*), while the magenta dashed lines indicate the Fermi level during xylene exposure ($E_{f}$ *in xylene*). The blue arrows indicate the proposed direction of the Fermi-level shift upon xylene exposure. The band positions and Fermi-level shifts are schematic and were not measured directly.

The proposed interaction between xylene and the hole-dominated DWNT network is illustrated schematically in Figure 9. The absolute conduction-band and valence-band positions and the magnitude of any Fermi-level shift during gas adsorption were not measured directly. Accordingly, the electronic structure shown in Figure 9 should be regarded as a qualitative representation rather than an experimentally determined band alignment. Xylene has been reported to behave as an electron-donating analyte toward CNT sensing layers, producing an n-type perturbation of an initially p-type nanotube network [42]. In a hole-dominated DWNT network, electron donation would partially compensate the majority holes, decreasing the effective hole concentration and therefore the conductivity. This interpretation is consistent with the experimentally observed increase in resistance during xylene exposure, for which $R_{g}>R_{a}$.

For pristine DWNTs, the smaller xylene response can therefore be represented schematically by a comparatively small perturbation of the effective Fermi level. In Cr-DWNTs, the same general charge-transfer process is proposed to occur against a different baseline electronic state. Because the Cr-DWNT begins with a lower apparent Hall hole concentration while remaining hole-dominated, electron donation associated with xylene adsorption may constitute a larger fractional perturbation of the available hole population. Adsorption at electronically sensitive Cr_2_O_3_–DWNT interfaces and modified CNT–CNT junctions may additionally alter local potential barriers and inter-tube transport, further contributing to the measured resistance change.

The overall sensing mechanism can therefore be described as a recognition–amplification process. The graphitic DWNT framework provides the underlying molecular recognition through its stronger interaction with the more highly methyl-substituted and polarizable xylene molecule, producing the intrinsic BTX response order xylene > toluene > benzene. Chromium modification then alters the baseline electronic transport state of the hole-dominated DWNT network and introduces additional electronically sensitive interfacial regions. Xylene adsorption consequently produces a larger fractional perturbation of the modified network than in pristine DWNTs. The combined effects of intrinsic xylene–graphitic affinity, adsorption-induced hole compensation, and chromium-associated electronic modification therefore provide a physically consistent explanation for the enhanced room-temperature xylene response and improved BTX discrimination observed for Cr-DWNTs.

### 4.5. Comparison with Reported Xylene Sensors

To place the performance of Cr-DWNTs in the context of previously reported xylene sensors, Table 3 compares representative sensing materials reported in the literature. The comparison includes both room-temperature and elevated-temperature devices because operating temperature is an important consideration for practical low-power sensing.

The comparison in Table 3 highlights the different performance characteristics of reported xylene sensors. Several metal-oxide sensors exhibit substantially larger numerical response values and rapid response/recovery kinetics; however, these measurements are generally obtained at elevated operating temperatures and at xylene concentrations substantially higher than those investigated in the present work. The Cr-DWNT operates at room temperature and exhibits measurable preferential xylene response down to 0.1 ppm, with a calculated LOD of approximately 37 ppb. The principal distinction of the present system is therefore the combination of room-temperature operation, low-ppm xylene response, and enhanced discrimination within the structurally similar BTX group. Although the Cr-DWNT offers the advantage of room-temperature sensing without continuous external heating during operation, preparation of the hybrid currently requires a high-temperature sealed-ampoule treatment at 854 °C for 24 h, followed by controlled cooling, washing, and freeze-drying. The preparation procedure is therefore more energy- and time-intensive than simpler solution-based or direct-deposition fabrication routes. Nevertheless, the high-temperature treatment is confined to material preparation, whereas the resulting sensor operates at room temperature without an external heater. Future optimization of the filling time, thermal-treatment conditions, and post-processing steps may improve the scalability and reduce the energy demand of the preparation procedure.

## 5. Conclusions

This study demonstrates the enhanced room-temperature low-concentration xylene-sensing performance of chromium-modified double-walled carbon nanotubes (Cr-DWNTs). Whereas pristine DWNTs showed broad responses toward several analytes and only weak separation between the BTX compounds, Cr-DWNTs maintained a preferential response toward xylene throughout the investigated 0.1–10 ppm concentration range. The enhanced xylene response is therefore proposed to arise from the combined effects of intrinsic xylene–DWNT affinity and chromium-induced electronic amplification of the DWNT network. Overall, these findings demonstrate the potential of Cr-DWNTs as a promising room-temperature chemiresistive material for preferential xylene detection. Its operation without external heating, together with its enhanced low-concentration response and improved BTX discrimination, highlights its potential for the development of low-power VOC sensing platforms for environmental monitoring and air-quality assessment, with possible future application in breath-analysis systems following validation under realistic humidity and mixed-gas conditions.

## Figures and Tables

**Table 1:** Hall-effect and electrical transport parameters of pristine DWNT and Cr-DWNT measured at room temperature.

Parameter	Pristine DWNT	Cr-DWNT
Hall coefficient, $R_{H}$ (cm^3^ C^−1^)	4.2533	7.9046
Carrier type	p-type	p-type
Apparent Hall carrier concentration, $p_{H}$ (cm^−3^)	$1.47\times 10^{18}$	$7.90\times 10^{17}$
Hall mobility, *μ*H (cm^2^ V^−1^ s^−1^)	$2.6399\times 10^{2}$	$1.1688\times 10^{3}$
Conductivity, σ (S cm^−1^)	$6.2068\times 10^{1}$	$1.4786\times 10^{2}$
Resistivity, ρ (Ω cm)	$1.6111\times 10^{-2}$	$6.7630\times 10^{-3}$

**Table 2:** Response and recovery times of pristine DWNTs and Cr-DWNTs toward the investigated VOCs at 1 ppm, determined using the $t_{90}$ criterion.

Analyte	Cr-DWNT		Pristine DWNT	
	$t_{90,\mathrm{res}}$ (s)	$t_{90,\mathrm{rec}}$ (s)	$t_{90,\mathrm{res}}$ (s)	$t_{90,\mathrm{rec}}$ (s)
Xylene	63.1	135.1	57.4	121.8
Toluene	64.8	60.6	42.1	95.2
Benzene	74.1	10.6	46.8	106.3
Methanol	73.1	118.0	57.6	126.0
Ethanol	64.1	106.4	33.3	80.1
Acetone	81.6	121.7	60.6	87.5
Pentane	9.0	180.0	9.2	35.4

**Table 3:** Comparison of the xylene-sensing performance of Cr-DWNTs with representative previously reported chemiresistive xylene sensors.

Sensing Type	Operating Temp. °C	Xylene Concentration	Reported Response	Response/Recovery Time (s)	Detection Capability	Reference
Au-WO_3_·H_2_O nanocubes	°C	5 ppm	26.4	∼1/∼1	∼200 ppb	[9]
NiO/NiCr_2_O_4_ nanoparticles	225 °C	100 ppm	66.2	1217/591	Response demonstrated at 50 ppb	[4]
Ru-WO_3_ nanosheets	280 °C	100 ppm	73	N.R.	∼25 ppb LOD	[43]
Cr-doped WO_3_ thin film	RT	50 ppm	6.4	12/7	Tested over 1–50 ppm	[25]
**Cr-DWNT**	**RT**	**0.1–10 ppm**	**0.96% at 0.1 ppm; 5.93% at 10 ppm**	**63.1/135.1 at 1 ppm**	**37 ppb calculated LOD**	**This work**

N.R.: not reported.

## Data Availability

The original contributions presented in this study are included in the article/Supplementary Materials. Further inquiries can be directed to the corresponding author.

## Supplementary Materials

The following supporting information can be downloaded at: https://www.mdpi.com/article/10.3390/ma19204378/s1, Figure S1: Bar graph comparison of the room-temperature responses of pristine DWNT and Cr-DWNT toward the investigated VOCs at (a) 0.1 ppm, (b) 1 ppm, (c) 5 ppm, and (d) 10 ppm. Bars represent the mean response of three replicate measurements (n=3), and error bars represent the corresponding standard deviation. Table S1: Selectivity factors of Cr-DWNT toward xylene relative to the investigated interfering analytes at different concentrations. Figure S2: Representative HRTEM image of pristine DWNT showing the characteristic bundled nanotube morphology prior to chromium modification. Scale bars correspond to 20 nm.
