# Supplementary material for: Chromium-Decorated Double-Walled Carbon Nanotubes for Preferential Room-Temperature Xylene Sensing
Source: Materials (Basel). 2026 Oct 18;19(20):4378. doi: 10.3390/ma19204378 (PMC13707281; doi:10.3390/ma19204378)
Supplement: Supplementary file 1 [file materials-19-04378-s001.zip › materials-4576470-supplementary.pdf]

materials

Article

Chromium-Decorated Double-Walled Carbon Nanotubes for Preferential Room-Temperature Xylene Sensing

Tlotlo Cassandra Setlhare [1], Atlang Gild Mpolokang [1], Emmanuel Flahaut [2], Martin Figueiredo [2] and George Chimowa [1,*]

1 Department of Physics and Astronomy, Botswana International University of Science and Technology, Boseja Ward, Palapye Private Bag 16, Botswana; st19100123@biust.ac.bw (T.C.S.); ma19100109@biust.ac.bw (A.G.M.)
2 Université de Toulouse, Toulouse INP, CNRS, CIRIMAT, 31062 Toulouse, France; emmanuel.flahaut@utoulouse.fr (E.F.); martin.figueiredo@utoulouse.fr (M.F.)
* Correspondence: chimowag@biust.ac.bw

Supplementary Information

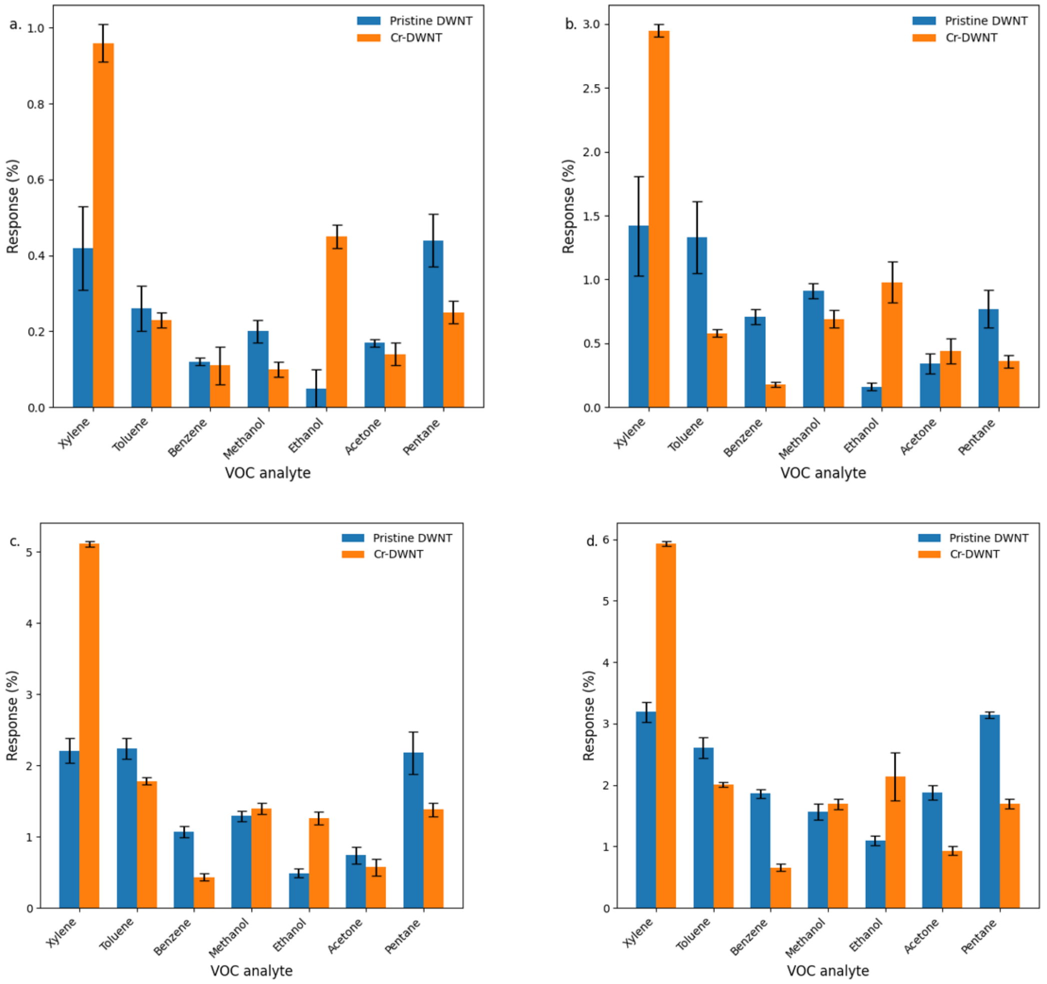

Figure S1. Bar graph comparison of the room-temperature responses of pristine DWNT and Cr-DWNT toward the investigated VOCs at (a) 0.1 ppm, (b) 1 ppm, (c) 5 ppm, and (d) 10 ppm. Bars represent the mean response of three replicate measurements ($n = 3$), and error bars represent the corresponding standard deviation.

Check for updates

Academic Editors: Yang Shi, Junjie Ye and Yongkun Wang

Received: 4 September 2026
Revised: 25 September 2026
Accepted: 29 September 2026
Published: 18 October 2026

Copyright: © 2026 by the authors. Licensee MDPI, Basel, Switzerland. This article is an open access article distributed under the terms and conditions of the Creative Commons Attribution (CC BY) license.

Table S1. Selectivity factors of Cr-DWNT toward xylene relative to the investigated interfering analytes at different concentrations.

Interferent	0.1 ppm	1 ppm	5 ppm	10 ppm
Toluene	4.17	5.09	2.87	2.95
Benzene	8.73	16.39	11.88	8.98
Methanol	9.60	4.27	3.65	3.51
Ethanol	2.13	3.01	4.06	2.76
Acetone	6.86	6.70	8.96	6.38
Pentane	3.84	8.19	3.70	3.49
Water	3.31	3.55	3.12	3.24

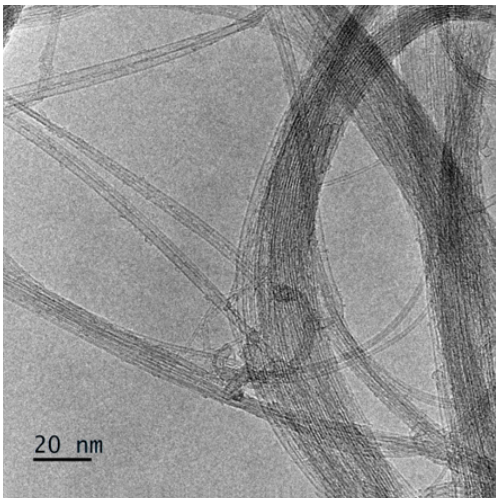

Figure S2. Representative HRTEM image of pristine DWNT showing the characteristic bundled nanotube morphology prior to chromium modification. Scale bars correspond to 20 nm.
